# Modeling the relapse distribution of *Plasmodium vivax* in different geographies

**DOI:** 10.1186/1475-2875-13-S1-P27

**Published:** 2014-09-22

**Authors:** Philip Eckhoff, Edward Wenger

**Affiliations:** 1 Institute for Disease Modeling, Bellevue, WA, USA

## 

One of the defining challenges of controlling and eliminating *Plasmodium vivax* is the tendency to relapse. Months or even years after clearing the initial infection, new blood-stage infections can emerge from hypnozoites in the liver if they are not cleared with radical cure. The relapse phenomenon is further complicated by the fact that *P. vivax* infections exhibit different patterns of relapse times in different areas. The tropical Chesson variant has a short distribution, with most relapses occurring in the first 3-4 months after initial infection. Other variants have an initial infection, early relapses, and then later relapses, 8-12 months, after the initial infection. Still others have relapses over a year after the initial infection, and some do not exhibit an initial infection at all. We construct a unified mathematical model for the distribution of three important patterns, demonstrate how each distribution is well-suited to the local transmission dynamics in which it is observed, and discuss the implications for control and elimination.

